# Decision-Making Scores and Hunger Susceptibility: A Positive Correlation Mediated by Fasting FGF21 Independently of Body Fat

**DOI:** 10.3390/nu17193160

**Published:** 2025-10-06

**Authors:** Andrés M. Treviño-Alvarez, Tomás Cabeza de Baca, Emma J. Stinson, Hannah T. Fry, Marci E. Gluck, Douglas C. Chang, Paolo Piaggi, Jonathan Krakoff

**Affiliations:** 1 Department of Health and Human Services, Obesity and Diabetes Clinical Research Section, Phoenix Epidemiology and Clinical Research Branch, National Institute of Diabetes and Digestive and Kidney Diseases, National Institutes of Health, Phoenix, AZ 85016, USA; trevinoalvarez.andres@mayo.edu (A.M.T.-A.); tommy.cabezadebaca@nih.gov (T.C.d.B.); emma.stinson@nih.gov (E.J.S.); gmarci@niddk.nih.gov (M.E.G.); douglas.chang@ihs.gov (D.C.C.); jkrakoff@mail.nih.gov (J.K.); 2 Department of Neurology, Universidad Autónoma de Nuevo León, Monterrey 66455, Mexico; 3 The Warren Alpert Medical School, Brown University, Providence, RI 02912, USA; 4 Department of Information Engineering, University of Pisa, 56126 Pisa, Italy

**Keywords:** FGF21, hunger, disinhibition, decision making, fasting

## Abstract

Background/Objectives: Understanding the relationship between metabolism and eating behavior may improve how we treat and prevent obesity. Fibroblast growth factor 21 (FGF21) is a hormone secreted by the liver with a putative role in energy expenditure, energy intake, and weight regulation. In this secondary analysis, we studied how fasting FGF21 is correlated with eating behavior and decision making, as measured by the Three-Factor Eating Questionnaire (TFEQ) and the Iowa Gambling Task (IGT), respectively. Methods: Participants (n = 98; women = 19; white = 31) were medically healthy, between 18 and 55 years of age, weight-stable 6 months before admission, and had normal glucose regulation. Women were premenopausal and not pregnant. Pearson partial correlations were determined, accounting for age, sex, and body fat percentage. A mediation analysis examining whether the association between hunger and IGT score was mediated by FGF21 values was performed using general linear models. Results: In partial correlations adjusted for age, sex, and body fat percentage, we found that fasting FGF21 concentrations were positively correlated with hunger susceptibility (sum of internal and external cues) (partial r = 0.26, *p* = 0.02) and internal hunger (partial r = 0.22, *p* = 0.04), disinhibition (partial r = 0.27, *p* = 0.01), and better decision making (higher IGT scores) (partial r = 0.40, *p* = 0.0001). We also found a correlation between hunger susceptibility and better decision making, including the same covariates (partial r = 0.25, *p* = 0.03). However, this correlation was mediated (36%) by fasting FGF21. Conclusions: In this study, participants with greater susceptibility to hunger cues had higher IGT scores (better decision making) in the setting of higher fasting FGF21 concentrations. This provides further evidence of the role of FGF21 in the interplay between eating behavior and decision making. Further studying this topic may improve our understanding of the complex relationship between assessing energy requirements and cognitive processes related to eating behavior.

## 1. Introduction

As obesity rates increase, so do the related medical and mental health consequences [1,2,3]. Improving obesity prevention strategies includes investigation of the complex interplay between metabolism and eating behavior [4]. Eating behavior is driven by different factors, including energy expenditure, hedonism, sociocultural experiences, and cognitive processes such as hunger susceptibility, disinhibition, restraint, and decision making [4,5]. Importantly, in humans, eating behavior also implies a choice made given an internal assessment of energy and macronutrient requirements. The complexity required to assess energy stores and determine the need for food consumption is highly regulated by the brain [6]. Conversely, brain and mental health alterations have also been correlated with both eating and metabolic disorders [3,7,8]. Thus, it is important to study the factors involved in the interplay between metabolism and behavior. One such candidate is fibroblast growth factor 21 (FGF21) [9].

FGF21 is a hormone well known for its role in metabolism. While affecting glucose, lipid, and bone metabolism [10,11], FGF21 has also been implicated in energy-consuming behavior. In animal models, FGF21 infusions decrease sugar and alcohol preference [12]. In obese mice models of cognitive decline, metabolic alterations, and FGF21 pathway alterations, treatment with FGF21 resulted in metabolic improvements and neuroprotective effects [13]. In humans, FGF21 increases in response to nutritional challenges [9], particularly 24 h of low protein overfeeding and binge alcohol drinking [14]. A lower increase in FGF21 concentrations following low protein overfeeding predicts weight gain [15], and higher fasting FGF21 is associated with less soda intake [16]. Hence, FGF21 has been thought to play a role in the interplay between identifying energy and specific macronutrient requirements and the cognitive process involved in choosing macronutrients, although more evidence is needed in humans [17]. Indeed, as with other hormonal analogues, pharmacologic use of FGF21 has been investigated as a potential intervention to change consuming behavior [9,18,19]. The role of FGF21 in human eating behavior is increasingly recognized as significant, particularly in the homeostatic regulation of macronutrient intake, energy balance, and feeding preferences [20]. FGF21 levels in circulation have been shown to correlate with several eating behaviors. Human cohort studies reveal that FGF21 concentrations rise in response to acute sugar and alcohol ingestion, suggesting that it acts as a regulatory hormone influencing cravings and food choices [14,21]. Specifically, after fructose ingestion, a significant increase in FGF21 levels was observed, indicating that FGF21 may play a nuanced role in the regulation of macronutrients, particularly under conditions of elevated glucose [21]. Additionally, studies illustrate that individuals who consume high-sugar diets tend to exhibit different FGF21-related metabolic profiles, supporting the hormone’s involvement in food preferences and dietary habits [22].

Given its potential dual role in weight regulation and cognitive function, we assessed the relationship between FGF21 concentrations and eating behavior and decision making using the Three-Factor Eating Questionnaire (TFEQ) and the Iowa Gambling Task (IGT). Individuals with obesity, with and without binge eating disorder, have lower IGT scores compared to controls [23]. We had previously found associations of lower IGT scores with higher body mass index (BMI) [24], and higher percent body fat, leptin, and insulin values [25]. We hypothesize that FGF21 may be associated with these measurements of cognition and eating behavior.

## 2. Methodology

This is a secondary analysis from a clinical trial (clinicaltrials.gov identifier: NCT00523627) that studied metabolic responses of healthy individuals exposed to 24 h of fasting and different overfeeding diets. The methodological details have been previously reported [26]. Here, we briefly describe the methodology relevant to this secondary analysis.

The study was carried out in the Clinical Research Unit of the National Institute of Diabetes and Digestive and Kidney Diseases (NIDDK) in Phoenix, Arizona, from 2008 to 2017. Enrolled individuals were between 18 and 55 years of age, medically healthy (assessed by medical history, physical exam, and general blood tests), weight-stable (body-weight variation <10% in the 6 months before admission), and weighing ≤ 450 lbs. Participants did not have a mental health diagnosis, nor were any on medications to treat a mental health disorder. Women (n = 19, 19.4%) were premenopausal and not pregnant. Exclusion criteria included impaired glucose tolerance (as determined by an oral glucose tolerance test [OGTT] following 3 days of a standardized weight-maintaining diet and characterized based on the American Diabetes Association’s glucose criteria [27]); history of type 1 or type 2 diabetes; current smokers; alcohol or drug abuse; and any history of cancer, cardiovascular, pulmonary, renal, endocrine, or infectious disease. The extensive exclusion criteria have already been published [28]. Informed consent was provided before starting the study. The study was approved by the IRB of the NIDDK (Study Protocol #: 07-DK-N215).

Of 183 participants screened, 98 met eligibility criteria, were admitted to the unit, and began a personalized weight-maintaining diet (50% carbohydrates, 30% fat, and 20% protein) [29]. Body weight was maintained within 1% of the admission weight. On day 2, a dual-energy X-ray absorptiometry (DXA) (Lunar Prodigy or iDXA [30]) (GE Lunar, Madison, WI, USA) was performed to assess body composition. DXA values were made comparable based on a published formula [30]. After day 3, a 75 g oral glucose tolerance test was performed, and only participants with normal glucose regulation continued the study [31]. Participants were given different diets (1 fasting and 5 overfeeding diets) for 24 h in the respiratory chamber (whole-room indirect calorimeter) with a 3-day washout period in between, during which time participants received the weight-maintaining diet. Blood samples were drawn before every dietary intervention, collected in EDTA-containing tubes, and stored in −70 °C freezers for later measurements. Fasting FGF21 concentrations were analyzed from these samples by ELISA (R&D Systems, Minneapolis, MN, USA). The intra-assay coefficient of variation (CV) was 2.5%, and the inter-assay CV was 5.2%. For each individual, all pre-diet fasting FGF21 measurements were averaged to calculate mean baseline concentration of fasting FGF21.

### 2.1. Cognitive and Behavioral Assessment

On day 2 of the study, participants answered self-reported behavioral questionnaires and neuropsychological computer tasks, administered 30 min after the morning meal to standardize timing with meals. All measures were previously validated and standardized in English and were only administered in English.

Three-Factor Eating Questionnaire (TFEQ) [5]: A 51-item questionnaire was employed to measure the following eating behaviors: cognitive dietary restraint (α = 0.84; 21 items), dietary disinhibition (α = 0.65; 16 items), and susceptibility to hunger (α = 0.80; 14 items). Cognitive restraint reflects the intent to restrict food intake to control body weight; disinhibition refers to the tendency to overeat; and susceptibility to hunger measures the inclination to eat in response to hunger cues (internal and external). The TFEQ has been extensively employed to study eating behavior [5,32].

Iowa Gambling Task (IGT) [33]: Participants were given a digital version of the test with four decks of cards (A, B, C, and D) from which they chose 100 cards, one at a time, resulting in winning or losing “money” in the game. Decks A and B were disadvantageous (large rewards, large penalties, overall net loss), and C and D were advantageous (small rewards, small penalties, overall net gain). IGT Net Total score results from the number of cards chosen from disadvantageous decks subtracted from the number of cards chosen from advantageous decks. IGT T scores and percentiles were calculated considering age and education variables by the IGT program. IGT Total Money reflects the amount of “money” won at the end. Positive scores reflect better decision making in this task. The IGT was originally developed to measure decision making in patients with alterations in the prefrontal cortex (PFC) by considering factor uncertainty, reward, and punishment [34]. This is highly relevant considering the role the PFC has in decision making in eating behavior [4,35]. The IGT is used in healthy individuals and those with disordered eating.

### 2.2. Statistical Analyses

Data analyses were performed using SAS software (SAS 9.3, Enterprise Guide version 5.1; SAS 9.4; SAS Institute Inc., Cary, NC, USA). The sample size for this secondary analysis was determined by data availability from the parent study. Continuous data are expressed as mean ± SD, except for data with skewed distribution, which are expressed as median with interquartile range (IQR). Categorical data are expressed as counts and percentages. An alpha less than 0.05 (two-tailed) was considered statistically significant. Pearson correlation coefficients quantified the associations between behavioral questionnaires, body composition, demographics, and FGF21. Additionally, partial correlation coefficients adjusted for age, sex, and body fat percentage were also calculated. A mediation analysis was performed to measure the role of FGF21 as a mediator of the correlation between IGT and Hunger (TFEQ) using the steps outlined by Baron and Kenny [36].

## 3. Results

### 3.1. Clinical Characteristics

Table 1 presents the demographic and clinical characteristics of our sample. Participants were mostly men (81%), with a mean BMI of 26.6 ± 4.3 kg/m^2^, and all were medically healthy (Table 1). Fasting FGF21 was positively correlated with BMI (r = 0.30, *p* = 0.004) and body fat percentage (r = 0.32, *p* = 0.001).

### 3.2. Correlations Between FGF21, IGT, and TFEQ

**Figure 1:**
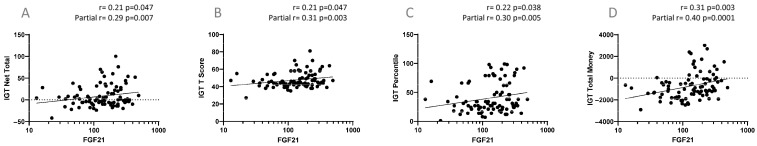
**Iowa Gambling Task scores and FGF21 correlations.** IGT, Iowa Gambling Task. The X axis represents fasting FGF21 concentrations in each graph; the Y axis varies according to different IGT scores: (**A**) IGT Net Total; (**B**) IGT T score; (**C**) IGT percentile; and (**D**) IGT Total Money. The lower section of the figure shows the correlation values after adjusting for age, sex, and body fat percentage.

**Figure 2:**
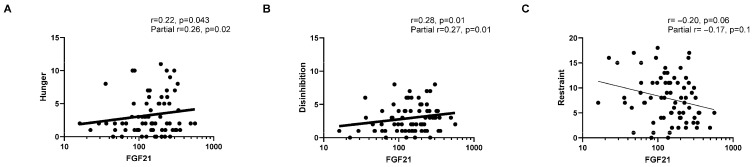
**Three-Factor Eating Questionnaire and FGF21 correlations.** The X axis represents fasting FGF21 concentrations in each graph; the Y axis represents the Three-Factor Eating Questionnaire categories: (**A**) hunger, (**B**) disinhibition, and (**C**) restraint. The lower section of the figure shows the correlation values after adjusting for age, sex, and body fat percentage.

**Figure 3:**
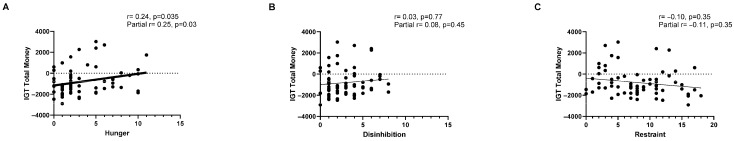
**Iowa Gambling Task Score and Three-Factor Eating Questionnaire correlations.** IGT, Iowa Gambling Task. The X axis represents the Three-Factor Eating Questionnaire categories: (**A**) hunger, (**B**) disinhibition, and (**C**) restraint; the Y axis represents the IGT T score. The lower section of the figure shows the correlation values after adjusting for age, sex, and body fat percentage.

Higher fasting FGF21 values were associated with higher IGT scores, with the strongest correlation with “Total Money” (Figure 1). Fasting FGF21 values were also correlated with higher scores of hunger susceptibility and disinhibition from the TFEQ (Figure 2A,B). IGT and TFEQ correlations with fasting FGF21 remained significant after adjusting for age, sex, and body fat percentage (IGT Total Money, partial r = 0.40, *p* = 0.0001; hunger, partial r = 0.26, *p* = 0.02; disinhibition, partial r = 0.27, *p* = 0.01). Simple correlations between fasting FGF21 and hunger subsets were not significant (internal hunger, r = 0.19, *p* = 0.08; external hunger, r = 0.18, *p* = 0.08), but the partial correlations were significant for internal hunger (internal, r = 0.22, *p* = 0.04; external, r = 0.18, *p* = 0.10). Figure 3 displays the correlations between IGT Total Money and TFEQ; only hunger was correlated and remained significant after adjustment (partial r = 0.25, *p* = 0.03).

### 3.3. Fasting FGF21 as a Mediator

**Figure 4:**
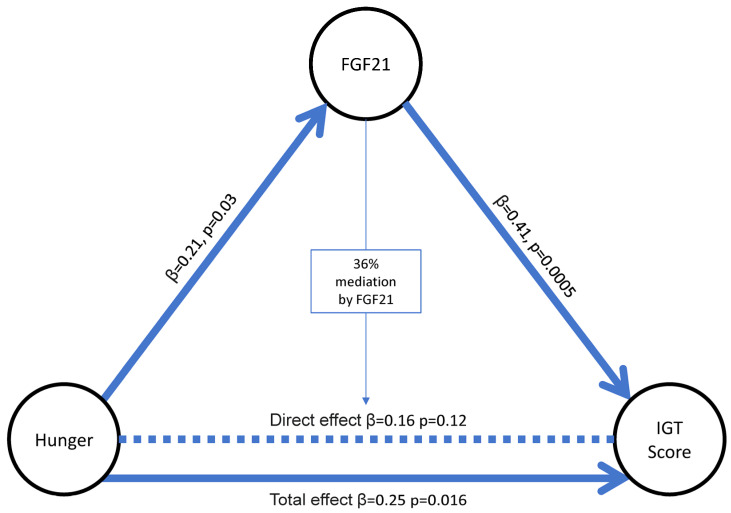
**Mediating role of FGF21 with hunger and decision-making scores.** Hunger, hunger perception from the Three-Factor Eating Questionnaire; IGT, Iowa Gambling Task. In this model adjusted for age, sex, and body fat percentage, Hunger had a significant total effect on IGT score (β = 0.25, *p* = 0.016). When adjusted for FGF21, the direct effect of hunger on IGT was not significant (β = 0.16, *p* = 0.12), demonstrating a 36% mediating role of FGF21. Full arrows represent significant correlations fulfilling the mediating criteria; the dotted line represents the direct effect without a significant *p* value.

A mediation analysis was performed to investigate how FGF21 might mediate hunger through its effect on decision making (Figure 4). The correlation between higher IGT scores and greater hunger was not significant when further adjusting for fasting FGF21. Hence, FGF21 mediated (36%) the correlation between greater IGT scores and greater hunger when adjusted for age, sex, and body fat %.

## 4. Discussion

In this study of healthy individuals, fasting FGF21 concentrations were positively correlated with better decision making (higher IGT scores), and higher hunger susceptibility and disinhibition (TFEQ), even after adjusting for age, sex, and body fat% (Table S1). Higher IGT scores and greater hunger susceptibility were also positively correlated after adjustment for the same covariates. However, this correlation was mediated by fasting FGF21. Here, higher fasting FGF21 in healthy individuals may represent an enhanced capacity to respond to energy needs.

Eating is a complex, motivated, consuming behavior that involves the interplay of factors related to energy homeostasis, hedonic signals, and sensory inputs, which are then regulated by brain executive functions to make eating decisions [4,35]. FGF21 may play a role in altering specific energy intake behavior [9]. FGF21 concentrations change in humans facing nutritional challenges such as prolonged fasting [37,38], protein restriction [39], overeating [40], high glucose intake [41], and binge alcohol drinking [14]. Animal models indicate that FGF21 induces dietary protein intake [9]. In a Danish cohort, FGF21 variants were associated with increased sweet food consumption, smoking, and alcohol intake [41]. In animal models receiving pharmacologic doses of FGF21, alcohol and sweet preference decreased [12], while higher fasting FGF21 concentrations in humans were correlated with less ad libitum soda intake [16]. Individuals with higher FGF21 concentrations demonstrated increased dislike for sugar [41]. In all the above cases, eating ultimately represents the decision to consume. Thus, our finding that higher fasting FGF21 is positively correlated to higher IGT scores indicates that FGF21 may play a role in the decision-making process that involves ingestive choices.

The IGT was originally developed to measure the degree of impaired decision making in regard to uncertainty, reward, and punishment in patients with damage to the ventromedial prefrontal cortex [34]. This assessment has subsequently been used in individuals with frank eating disorders and in those with milder degrees of disordered eating. Individuals with anorexia nervosa and higher IGT scores show greater improvement after treatment [42]. Lower IGT scores have been associated with higher body mass index (BMI) [24], higher waist circumference [43], higher body fat percentage, leptin, and insulin values [25]. FGF21 crosses the blood-brain barrier in animal models [44] and is found in human cerebrospinal fluid (CSF) [45]. Plasma and CSF FGF21 were correlated (r = 0.43, *p* < 0.01), and as in our analysis, both concentrations were also positively correlated with BMI and fat mass [45]. Even though an increase in adiposity has been hypothesized to reflect FGF21 resistance [46] and lead to dysfunctional FGF21 brain signaling [45], once adiposity was accounted for in our analysis, FGF21 appeared to have a salutary modulating effect on decision making.

In animal models, FGF21 receptors (FGFRs and KLB coreceptor) are expressed in brain regions that include the suprachiasmatic nucleus of the hypothalamus (linked to circadian rhythm activity) and the dorsal vagal complex of the hindbrain (linked to energy-sensing mechanisms) [47]. More recently, a subpopulation of KLB-expressing neurons was identified in the basolateral amygdala projecting to the nucleus accumbens in the striatum and found to play a role in decreasing alcohol intake in animal models receiving exogenous FGF21 [48]. Of interest is the finding that the amygdala is also considered an active site in decision making [49]. In our study, FGF21 was the mediator between higher scores in decision making and reporting greater hunger susceptibility, so we speculate that plasma FGF21 concentrations reflect central nervous system concentrations and facilitate communication between energy needs and eating behavior.

Hunger assessed by the TFEQ describes the susceptibility to internal or external hunger signs. Greater hunger scores from the TFEQ are associated with less activity at the left insular cortex—a region that integrates perception, cognition, and interoceptive awareness—in persons with and without obesity [50]. A study from the Sorbs Cohort employing a German version of the TFEQ found that FGF21 serum concentrations are associated with eating disinhibition [51]. We also found that greater disinhibition was associated with higher FGF21 concentrations, as well as with greater hunger susceptibility. Given the evidence that higher FGF21 concentrations appear to inhibit intake of sweet foods and soda, the positive association with hunger susceptibility in our study appears paradoxical [52]. However, hunger susceptibility as measured by the TFEQ can be considered to represent the appropriate anticipation of energy intake based on homeostatic need (internal hunger sign). Indeed, the positive association between IGT scores (as a measure of appropriate decision making) and hunger susceptibility appears to reinforce this. FGF21 may affect anticipatory energy intake by influencing decision making regarding the intake of selected foods and ensuring it is appropriate to the energy balance status of the individual.

Regarding limitations, there were relatively few women; the IGT assessment considers money as the assessment of the reward system, not food; and the TFEQ examined eating behavior experience as recalled by the participants. Further, the recruitment period was from 2008 to 2017. This may have introduced temporal and contextual biases because participant characteristics, external conditions, and minor procedural details can vary over time. Although we standardized study protocols and calibration procedures to mitigate variability, residual heterogeneity due to the long enrollment period cannot be excluded. Future studies should address this issue by implementing tighter recruitment periods or explicitly controlling for time. Despite our cohort not being large enough to reliably support complex multivariable models, we attempted a more comprehensive path analysis using structural equation modeling. In this analysis, we treated sex and age as exogenous variables, cognitive performance on the Iowa Gambling Task and fasting FGF21 as mediators, and BMI and eating behavior as outcomes, with TFEQ subscales considered as indicators of a latent eating construct. We employed procedures appropriate for small samples [53,54], including robust maximum likelihood, bootstrap confidence intervals, and constrained parameterization. Model fit was poor to modest, and several path estimates were imprecise, which is consistent with limited power. Future studies with larger cohorts, preferably longitudinal, should formally test this fuller network of direct and indirect relationships and the latent structure of eating behavior. Our study also has several strengths, including a diverse cohort, standardization of timing for completion (with respect to meals) of the measure of TFEQ and IGT, multiple measures of fasting FGF21 collected during a carefully controlled inpatient study, and the use of the DXA scan for body composition assessment.

## 5. Conclusions

In this study of healthy participants, fasting FGF21 values were correlated with greater hunger perception, eating disinhibition, and higher decision-making scores. A correlation between greater hunger perception and decision making was also found, and FGF21 mediated this relationship. Given the metabolic roles of FGF21, this evidence indicates the role of FGF21 in the intercommunication between energy needs and appropriate consuming behavior in response to these needs.

## Figures and Tables

**Table 1:** Demographic and clinical characteristics.

	Total n = 98
Women, n (%)	19 (19.4)
Age (years), mean ± SD, n = 98	37.0 ± 10.6
Race/Ethnicity, n (%)	
Black	22 (22.5)
White	31 (31.6)
Hispanic	15 (15.3)
Native American	30 (30.6)
Body weight (kg), mean ± SD, n = 98	79.6 ± 13.8
BMI (kg/m^2^), mean ± SD, n = 98	26.6 ± 4.3
Fat mass (kg), mean ± SD, n = 98	23.3 ± 10.3
Fat-free mass (kg), mean ± SD, n = 98	56.3 ± 9.6
TFEQ Disinhibition, mean ± SD, n = 95	3.2 ± 2.3
TFEQ Hunger, mean ± SD, n = 95	3.4 ± 3.1
TFEQ Restraint, mean ± SD, n = 95	8.0 ± 4.6
FGF21 levels (pg/mL), median [25 pct–75 pct], n = 95	152.0 [87.25–238.75]
Iowa Gambling Task scores, n = 89	
Raw scores	8.9 ± 25.5
Demographically corrected T score	47.7 ± 8.9
Demographically corrected percentile	40.8 ± 26.5
Total Money	−717.5 ± 1221

BMI, body mass index; TFEQ, Three-Factor Eating Questionnaire. Demographically corrected variables of the Iowa Gambling Task scores are adjusted for age and sex by the Iowa Gambling Task program. Because FGF21 had a skewed distribution, values are reported as the median and the interquartile range (25th and 75th percentiles).

## Data Availability

Data described in the manuscript, code book, and analytic code will be made available upon request pending application and approval.

## Supplementary Materials

The following supporting information can be downloaded at: https://www.mdpi.com/article/10.3390/nu17193160/s1, Table S1: Correlation Matrix of Study Variables of Interest.
