# Supplementary material for: Decision-Making Scores and Hunger Susceptibility: A Positive Correlation Mediated by Fasting FGF21 Independently of Body Fat
Source: Nutrients. 2025 Oct 6;17(19):3160. doi: 10.3390/nu17193160 (PMC12525705; doi:10.3390/nu17193160)
Supplement: Supplementary file 1 [file nutrients-17-03160-s001.zip › nutrients-3861513-supplementary.pdf]

Supplemental Table S1. *Correlation Matrix of Study Variables of Interest.*

	1	2	3	4	5	6	7	8
1. Log_{10} (FGF21)	--	0.20	0.18	-0.15	**0.43**	**0.24**	**0.30**	**0.30**
2. Disinhibition	**0.25**	--	**0.58**	0.12	0.18	0.02	0.05	0.05
3. Hunger	**0.21**	**0.62**	--	**-0.21**	**0.30**	**0.28**	**0.29**	**0.29**
4. Cognitive Restraint	-0.18	0.10	**-0.26**	--	-0.07	-0.03	-0.03	-0.03
5. IGT Total Money	**0.32**	0.15	**0.25**	-0.08	--	**0.74**	**0.71**	**0.71**
6. IGT Net Total	**0.21**	0.01	0.20	-0.06	**0.87**	--	**0.96**	**0.96**
7. IGT T Score	**0.24**	0.01	0.19	-0.08	**0.85**	**0.98**	--	**1.00**
8. IGT Percentile	**0.21**	-0.02	0.17	-0.05	**0.82**	**0.96**	**0.97**	--

Bolded correlation coefficients denote statistically significant associations ($p < 0.05$). Correlation coefficients below diagonal are unadjusted correlation coefficients. Correlation coefficients above the diagonal are partial correlations adjusted for age, sex, and percent fat.
